# Improving the maternity experience for Black, African, Caribbean and mixed-Black families in an integrated care system: a multigroup community and interprofessional co-production prioritisation exercise using nominal group technique

**DOI:** 10.1136/bmjqs-2024-017848

**Published:** 2024-11-27

**Authors:** Sarindi Aryasinghe, Phoebe Averill, Carole Waithe, Susan Ibuanokpe, Rhianna Newby-Mayers, Nawal Lakhdar, Moussa Amine Sylla, Elizabeth Cox, Sabrina Das, Erik Mayer

**Affiliations:** 1 NIHR Imperial Biomedical Research Centre, Imperial College London, London, UK; 2 NIHR North West London Patient Safety Research Collaboration, Institute of Global Health Innovation, Imperial College London, London, UK; 3 Lived Experience Expert, Imperial College London, London, UK; 4 Mamas House CIC, London, UK; 5 Apricot Wellbeing CIC, London, UK; 6 Listen to Act, London, UK; 7 London Borough of Hammersmith and Fulham, London, UK; 8 Obstetrics and Gynaecology, Imperial College Healthcare NHS Trust, London, UK

**Keywords:** womens health, patient-centred care, obstetrics and gynecology, healthcare quality improvement, health services research

## Abstract

**ABSTRACT:**

**Background:**

Ethnic inequities in maternity care persist in England for Black, African, Caribbean and mixed-Black heritage families, resulting in poorer care experiences and health outcomes than other minoritised ethnic groups. Co-production using an integrated care approach is crucial for reducing these disparities and improving care quality and safety. Therefore, this study aimed to understand the alignment of health and local authority professional perspectives with community needs on how to improve maternity experiences for this ethnic group within a London integrated care system (ICS).

**Methods:**

Between March and June 2024, five workshops were conducted with health professionals, local authorities, voluntary, community and social enterprise (VCSE) sector and the public from Black, African, Caribbean and mixed-Black heritage backgrounds across the North West London ICS. Using the nominal group technique (NGT), attendees prioritised ideas to improve the experience of maternity care for families from Black, African, Caribbean and mixed-Black heritage backgrounds, which were thematically synthesised using framework analysis.

**Results:**

Fifty-four attendees, covering primary, secondary, regional and national health professionals, public health teams from three local authorities, VCSE sector and the public, generated 89 potential interventions across 11 themes. All attendees prioritised improving staff knowledge and capacity in culturally competent care and communication. Community-identified needs for advocacy mechanisms and mental health support throughout the maternity pathway were not reflected in professional priorities.

**Conclusion:**

The study highlights the need for an integrated, community-centred approach beyond hospital settings when addressing ethnic inequities in maternity care, recognising key differences between community and professional priorities within an ICS. Leveraging lived experience expertise to lead the NGT community workshops was essential in building trust and buy-in of the overall prioritisation process.

**WHAT IS ALREADY KNOWN ON THIS TOPIC**

- Black, African, Caribbean and mixed-Black heritage families experience disparities in maternal health outcomes and care, with few proven interventions addressing ethnic inequities in maternity care.
- Co-production with healthcare professionals, local authorities and communities using an integrated care approach is key to reducing maternal health disparities, but understanding the full range of priorities for all relevant groups may be challenging.

**WHAT THIS STUDY ADDS**

- Communities and professionals prioritise culturally competent care and communication-related interventions, but community needs for stronger advocacy mechanisms and mental health support were not reflected among professional priorities.

**HOW THIS STUDY MIGHT AFFECT RESEARCH, PRACTICE OR POLICY**

- Involving community engagement experts and facilitators with lived experience is crucial for establishing trust and gaining buy-in when undertaking quality improvement efforts to address ethnic health inequities.
- The nominal group technique is a pragmatic, methodologically rigorous way of generating co-production priorities, while also highlighting potential knowledge gaps and differences in priorities between health professionals and service users.

## Introduction

 Addressing inequalities in maternity care is a global and national priority in England, with women and birthing people from minoritised ethnic communities in high-income countries facing poorer care experiences and health outcomes.^1^,^3^ Racial bias embedded at the organisational level and within interpersonal interactions with care professionals can lead to mistrust and individual sense of loneliness during care.^4^,^6^ Women from minoritised communities are also more likely to have inadequate maternity care information because of their care provider’s limited time, knowledge and misconceptions about women’s needs and preferences. They are also less likely to experience shared decision-making in planning for their care and are given less opportunities to express preferences as a result of these barriers and past negative experiences with care.^7 8^

In particular, women from Black, African, Caribbean and mixed-Black heritage backgrounds face disproportionate inequities in access, experience and outcomes of maternity care compared with other ethnic groups.^3 6 9 10^ A recent UK survey in England exploring the experiences of women and birthing people from this group showed that negative interactions with healthcare professionals (eg, being dismissed, use of discriminatory language, racial stereotypes) led to long-term emotional and psychological outcomes, shaping their perceptions and future engagement with maternity care. An example of this is the stereotyping of pain tolerance in Black, African, Caribbean and mixed-Black heritage women, which can lead to dismissal of pain and lack of appropriate pain relief.^11^ The 2023 UK parliamentary report on Black maternal health also highlighted the role of racism in driving maternal health inequalities,^12^ resulting in differentially allocated resources and fuelling underlying social determinants of poorer health.^4 13^ Despite this growing body of evidence, many interventions targeted towards improving maternity care in the UK are not explicitly aimed at reducing ethnic inequalities in the care provided.^14^ These findings demonstrate entrenched shortfalls in the safety and quality of care provided, contributing to a continuous trend of maternal mortality rates of three times that of their White counterparts in the UK.^10^

Maternity care in England is provided by multiple health and social care organisations, fragmented across a complex health system, and as such women report feeling they are navigating a technocratic system.^15^ To address these system complexities, integrated care systems (ICS) were established in 2022.^16^ The ICS provides a framework for National Health Service (NHS) organisations and social care providers (eg, local authorities) to collaborate with communities, aiming to enhance population health, reduce health disparities, boost productivity and foster socio-economic development.^16 17^

Given the longstanding ethnic disparities in care and outcomes, statutory organisations like the NHS and local authorities must collaborate with the voluntary, community, social enterprise (VCSE) sector and communities to build trust and implement meaningful, sustainable service improvements.^417^,^19^ In developing service improvements that address ethnic inequities in maternity, it is essential to operate from a community-informed perspective and use collaborative decision-making processes that promote local ownership.^20^ Co-production is an increasingly recognised approach to achieving this, in which professionals work in partnership with patients, carers and communities to plan, design and evaluate services to meet the needs of local populations.^18 21^ Constructive co-production within integrated care requires a clear focus,^22^ with agreement on shared goals and objectives.^23^ Challenges may arise from differences in public versus organisational priorities; in centrally coordinating and implementing solutions; ensuring the diversity and representation of public voices and bringing together lived experience and professional knowledge and perspectives.^24^ Given these complexities, co-production priorities must be jointly set, incorporating different perspectives through collaborative multiprofessional and community engagements.

Analysis of local data on maternity experiences in the North West London (NWL) ICS also reflects the national challenges in maternity care for Black, African, Caribbean and mixed-Black heritage families. For example, not being listened to by staff, not receiving enough information and lack of support during the postnatal period were factors that led to negative experiences of care.^25^ Additionally, routine electronic health record data analysis of antenatal booking data at a multisite acute NWL NHS Trust providing maternity services has shown that mothers from Black African backgrounds were significantly more likely to access antenatal care later than other ethnic groups.^26^

Therefore, prioritisation workshops with health professionals, public health teams within local authority, VCSE sector and members of the public were undertaken to understand alignment of professional perspectives with community needs in the NWL ICS in improving experience of maternity care for Black, African, Caribbean and mixed-Black heritage families. As positive experiences of care are associated with improved safety and health outcomes,^27^ we sought to co-produce local solutions to inform potential interventions aimed at improving experiences of maternity care for this minoritised ethnic group.

## Methods

### Design

Nominal group technique (NGT) was used to elicit priorities and reach consensus, involving the following steps: silent individual idea generation, round robin (when each attendee presents one idea in turn), discussion for clarification and individual ranking. NGT identifies problems and solutions,^28^ equalises group dynamics by ensuring the voices of all attendees are heard^29 30^ and facilitates comparisons of priorities between groups.^31^ Study reporting corresponds to the Accurate Consensus Reporting Document (ACCORD) guidelines for consensus studies.

#### Eligibility criteria

Due to the complexity of the maternity care pathway within the ICS, five workshops were held: three with ICS professionals (NHS staff and three NWL local authorities) and two with community members and the VCSE sector. Eligibility criteria per workshop are detailed in table 1.

**Table 1:** Eligibility criteria for NGT workshops

Workshop	Inclusion criteria	Exclusion criteria
Maternity hospital staff (nominal group 1)	Currently employed within host multisite acute teaching hospital that provides maternity services. Works directly in maternity care, health inequalities, the organisation’s EDI strategic work or another related field.	Not employed within the host acute teaching hospital. Not working directly or related to maternity care, health inequalities, EDI work or another related field.
Wider NHS staff (nominal group 2)	Currently employed within the NHS in NWL in a primary care or regional role. Currently employed within the NHS in a national role contributing to maternity policy.	Not currently employed within the NHS in NWL in a primary care or regional role, or in a national maternity policy role.
Local authority (nominal group 3)	Currently employed or provide commissioned services for Hammersmith and Fulham, Kensington and Chelsea and Westminster City local authorities. Works directly or related to maternity care, public health and social care service evaluations and research.	Not exclusively employed or receiving commissioned funding from Hammersmith and Fulham, Kensington and Chelsea and Westminster City local authorities. Do not work directly or related to maternity care, public health and social care service evaluations and research.
Community and VCSE sector (nominal groups 4 and 5)	At least 18 years of age. Previous maternity service users at the host acute hospital, particularly those from Black, African, Caribbean and mixed-Black heritage backgrounds. Current residents of Hammersmith and Fulham, Kensington and Chelsea and Westminster City NWL boroughs. OR Member of a VCSE organisation that provides maternity support for families from Black, African, Caribbean and mixed-Black heritage families living in Hammersmith and Fulham, Kensington and Chelsea and Westminster City boroughs.	Under 18 years of age Not previous maternity service users or without experience of providing Black, African, Caribbean and mixed-Black heritage families with maternity support. Do not live in Hammersmith and Fulham, Kensington and Chelsea and Westminster City boroughs.

EDI, equality, diversity and inclusion; NHS, National Health Service; NWL, North West London; VCSE, voluntary, community, and social enterprise.

#### Workshop recruitment process

Purposive and snowballing recruitment approaches were used, leveraging author contacts in NHS, local authority, and voluntary sector organisations working in Hammersmith and Fulham, Kensington and Chelsea and Westminster City boroughs. Public recruitment was conducted through volunteer maternity and family champions networks operating in the three boroughs. Public-facing flyers invited interested individuals to register their attendance and indicate language and accessibility requirements. Registration was capped at 16 attendees per workshop, with drop-offs expected, ultimately aiming for 5–10 attendees as recommended in NGT literature.^32^

### Data collection

Separate workshops were conducted to understand the alignment of ICS professionals’ priorities with community needs for improving experience of maternity care for Black, African, Caribbean and mixed-Black heritage families.

#### Conduct of the health and local authority NGT workshops

Ninety-minute staff workshops were held online (Microsoft Teams) and chaired by the lead author. Before each workshop, session objectives and the discussion question were shared: “What needs to change to improve the experience of maternity care for women and pregnant people from Black, African, Caribbean and mixed-Black heritage backgrounds?” Participants were prompted to consider each stage of the maternity pathway (antenatal, childbirth and postnatal care). After reviewing workshop objectives, verbal consent was obtained. Ideas were recorded live using Miro, with duplicates grouped during the round robin and clarification phase. Attendees then ranked their top five ideas by importance (1—most important and 5—least important), and provided demographic details in an anonymous online (Qualtrics) survey. All ideas and group rankings were shared postworkshop with attendees. Table 2 describes the workshop structure.

**Table 2:** Structure of the NGT workshops

	Health and local authority NGT workshops(90 min)	Community NGT workshops(2.5 hours)
*Introduction*: icebreaker, overview of project and session objectives, setting of ground rules.	10 min	20 min
*Initial discussion sharing experiences of using maternity services*: to create a safe, supportive space, attendees of the community workshops were invited to share their experiences using maternity services in small groups if they felt comfortable.	N/A	30 min
*Silent generation of ideas*: attendees recorded all possible ideas individually in response to prompt question.	10 min	10 min
*Round robinandclarification (combined)*: each attendee presents one idea in turn until all ideas are exhausted. Clarifications happened organically at the same time.	15 min	30 min
*Sharing of ideas to full group*: ideas from each table were shared with the room, which were recorded in an online survey simultaneously.	N/A	25 min
*Voting and ranking*: attendees select their top five preferences and rank them (ie, 1=most important, 5=least important).	10 min	10 min
Wrap-up and feedback	5 min	10 min

N/A, not available; NGT, nominal group technique.

#### Conduct of the community NGT workshops

Workshop agendas, flyers and information sheets were developed and revised by all study authors, which included NHS and local authority professionals, as well as lived experience experts from Black, African, Caribbean and mixed-Black heritage backgrounds. Workshops took place over 2.5 hours in community centres, led by two independent community engagement specialists of Black African heritage, experienced in running trauma-informed community consultations. The approach promoted a holistic, strengths-based collaborative discussion and thus focused on deriving solutions that would improve maternity care experiences, recognising the impact of traumatic healthcare experiences and enabling supportive and empowering connections between individuals.^33^ Workshop attendees were organised into subgroups of four to five members. Lived experience expert coauthors facilitated each subgroup, having received prior training by the two community engagement specialists on trauma-informed facilitation skills. They were reimbursed for their involvement according to the National Institute for Health and Care Research (NIHR) public contributor payment guidance.^34^ Notetakers summarised each subgroup discussion on flipchart paper.

Workshops began with reviewing session objectives and collaborative setting of ground rules, including the option for attendees to leave at any time. Subgroup discussions commenced once attendees had agreed the ground rules and provided verbal informed consent. To build trust and promote a safe space, attendees were first invited to share their experiences of maternity care if they felt comfortable. Next, each subgroup progressed through the NGT steps using the question: “What could be done to improve the experience of your maternity journey?” VCSE leads and community champions, if not of Black, African, Caribbean and mixed-Black ethnicity themselves, were asked to answer the question from their experiences supporting families from those ethnic backgrounds. Following the clarification phase, subgroups shared their ideas with the lead facilitators, which were entered live into an anonymous online (Qualtrics) survey. Using this survey, attendees ranked their top five ideas from one (most important) to five (least important). Group rankings were emailed to attendees after the workshop, along with a £10 Amazon voucher, an amount previously agreed on with community champion borough managers to align with existing practices.

### Data analysis

Analyses involved three concepts: ‘ideas’ (generated during the silent generation and round robin), ‘priorities’ (top five ideas ranked by workshop attendees) and ‘themes’ (generated from the thematic analysis of ideas to support comparisons between nominal groups, and which contain relevant subthemes).^35^ Data analysis encompassed four steps: (i) quantitative analysis of individuals’ ranked ideas to generate priorities; (ii) thematic analysis of ideas to generate themes; (iii) ranking of each group ideas by theme and (iv) detailed framework analysis of workshop meeting notes using NVivo, focusing on the top five priority themes raised by community members. Steps 1–3 were conducted in Microsoft Office Excel.

#### Step 1: analysis of individuals’ ranked ideas to generate priorities

Within each nominal group, total scores were calculated by summing individual rankings for each idea (online supplemental file 1). Individuals’ highest ranked ideas received five points, and the fifth highest received one point. For the top five ideas, the relative importance (ie, the percentage of all scores in the top five) was calculated using the equation: [total score for the idea/(number of attendees in the group×total possible scores (ie, 5+4+3+2+1)]×100.^35 36^ If the total score for an idea was 30 points, and the nominal group had six participants, then the relative importance percentage would be calculated by: [30/(6×15)]×100. If multiple ideas received the same score, the number of times the idea was prioritised by attendees determined the final ranking. For example, if two ideas received the same relative importance score, but one was prioritised five times and the other was prioritised three times, the former idea would receive a higher overall ranking.

#### Step 2: thematic analysis of ideas to generate themes

Using methodology proposed by McMillan *et al*,^35^ ideas generated by the nominal groups were thematically analysed by the lead author, generating a coding framework. This was informed by a rapid literature review, workshop meeting notes and lead author’s professional experience in reproductive health and patient experience research and programme implementation. The initial coding framework was reviewed independently by two coauthors with qualitative research and maternity care clinical expertise, and subsequently revised to generate a final framework with 11 overarching themes and 28 subthemes. Ideas generated by the five nominal groups were then coded according to this framework (table 3).

**Table 3:** Thematic framework derived from nominal group ideas

Theme	Description	Subthemes
Respect for person-centred care	Treating service users with dignity and considering their unique needs, preferences and values, *for example, shared decision-making during appointments, listening to mother’s needs and respecting birth plans, respecting body autonomy.*	Listening to service user needs Respecting patient choice Informed consent
Information and education	Providing service users with better quality and tailored information and education, *for example, face-to-face health education appointment for all mothers that is separate to ANC visits, improving quality of patient information on websites, leaflets, social media.*	Quality of information provided Availability and quality of translation and interpretation services
Advocacy mechanisms	Strategies to support service users to assert their rights and voice their concerns, *for example, community advocates working within hospital settings, raising awareness of birthrights resources, improving feedback loop within complaints process.*	Use of community advocates Supporting service users to advocate for themselves Improving transparency and use of complaints process
Mental health	Mental health support during antenatal, labour/childbirth and postnatal period, *for example, counselling and emotional support during pregnancy, personalised and culturally appropriate mental health support, mental health teams supporting both parents.*	Mental health support for mothers Mental health support for husbands and partners
Involvement of friends and family	Involving immediate support network of service users in all stages of care and when improving services*, for example, tailored antenatal classes for Black fathers, using Black male NHS health professionals as role models to support Black fathers.*	Involving men and partners at all stages of care Support for birth partner selection
Access	Accessing of care, *for example, out-of-hours support, earlier uptake of first ANC appointment.*	Limited access Sustaining continued patient engagement with services
Staff knowledge and capabilities	Training and support for staff to deliver quality care for Black families*, for example, increasing cultural competency of workforce through mythbusting and cultural safety training and diverse recruitment, improving staff to patient ratios.*	Culturally competent care training Communication training Staff health and well-being
Ethnicity-based safety risks	Increased education and awareness of ethnicity-based patient safety risks, *for example, staff awareness of safety risks specific to service users from Black heritage groups.*	Identifying patient safety warning signs (eg, vital signs) Reducing ethnic biases in medical devices
Organisational policies	Initiatives and policies that are governed by organisational structures, processes and culture, *for example, prioritisation of EDI values, shared learning system for safety incidents related to Black mothers, zero tolerance policy for discrimination towards patients.*	Learning safety culture Staff disciplinary measures Improve data collection and monitoring Culturally appropriate catering
Community partnerships	Building trusted relationships with communities to improve services*, for example, sustained engagement with communities by maternity hospital staff, professional-community networking events to understand needs.*	Community engagement Community co-production of services
Coordination and integration of care	Communication and collaboration between organisations within an integrated care system*, for example, continuity of midwifery care, clearer signposting to non-health-related resources (housing, economic support), partnerships with community and schools to increase maternal health knowledge and engagement at early age*.	Continuity of care and carer Integrated system working (health, local authority, voluntary sector, community) Intersectional and holistic support for families Neonatal intensive care experience for parents

ANC, antenatal care; EDI, equality, diversity and inclusion; NHS, National Health Service.

#### Step 3: analysis of the ideas to generate priority themes

Total scores (sum of all points) for each theme were then calculated by nominal group (maternity hospital staff; wider healthcare staff; local authorities and members of the public and voluntary sector). The percentage relative importance score was computed, reflecting the total value placed on a theme by each group (ie, total score for theme/number of attendees in the group×maximum possible score (ie, 5+4+3+2+1)×100). For example, the theme ‘information and education’ received 54 points within the six-attendee maternity hospital staff group (so [54/(6×15 points)×100]). As with the generation of priorities from individual ideas, if two themes received the same percentage relative importance, the frequency each theme was voted was used to determine the final ranking (online supplemental file 2).

#### Step 4: framework analysis of meeting notes

Finally, framework analysis^37^ of workshop meeting notes was conducted to generate a detailed contextualisation of priority themes. This was done by the lead author first familiarising with meeting notes, and subsequently systematically applying the thematic framework developed in table 3. This was then reviewed and discussed by the other authors to finalise interpretation from health professional, researcher and community perspectives. As the ambition of co-production in service transformation is to address community needs, the qualitative analysis focused on the top five themes generated by the community groups. Consensus or diverging views between the community and professional groups within these five themes were also reviewed.

## Results

### Demographics

Healthcare and local authority workshops were held from March to May 2024, with community workshops in June 2024. Fifty-four individuals attended one of five NGT workshops, with 38 (70.3%) remaining until the end of the session to complete the ranking survey. Of these, half were members of the public and VCSE leads. 30% of the attendees that could not complete the survey were health professionals, who had to leave the workshop early due to conflicting meetings or clinical duties.

Data were not collected from individuals that did not complete the ranking survey. Most attendees were female (92%, n=35), and 57.8% (n=22) were from minoritised ethnic backgrounds. In the community nominal groups, 63.1% (n=12) of attendees were from Black, African, Caribbean and mixed-Black heritage backgrounds. To maintain attendee anonymity, only essential demographic data were collected, given the small number of attendees in each workshop and the sensitivity of some community participants reflecting on their maternity experiences for the very first time (table 4).

**Table 4:** Demographics of nominal group attendees

	Attendees in health and local authority nominal groups (n=19)	Attendees in community nominal group(n=19)
Professional profile		
Local (community, primary, secondary)	6 (31.6%)	–
Integrated care board	4 (21.1%)	–
National maternity policymakers	4 (21.1%)	–
Local authority	5 (26.3%)	–
Ethnicity		
Asian, Asian British or Asian Welsh	2 (10.5%)	–
Black, Black British, Black Welsh, Caribbean, African and any other Black background	7 (36.8%)	11 (57.9%)
Mixed/Multiple ethnic groups (Black African, White)	1 (5.3%)	1 (5.3%)
White: English, Welsh, Scottish, Northern Irish or British	6 (31.6%)	–
White: Irish	1 (5.3%)	–
White: Gypsy or Irish traveller, Roma or other White		2 (10.5%)
Other ethnic group	–	2 (10.5%)
Prefer to self-describe	–	2 (10.5%)
Prefer not to say	3 (15.9%)	1 (5.3%)
Gender		
Female	15 (94.7%)	18 (94.7%)
Male	1 (5.3%)	–
Non-binary	–	–
Prefer to self-describe	–	–
Prefer not to say	3 (15.9%)	1 (5.3%)
Age (years)		
18–24	–	–
25–39	2 (10.5%)	11 (57.9%)
40–49	7 (36.8%)	7 (36.8%)
50–79	7 (36.8%)	–
Prefer not to say	3 (15.9%)	1 (5.3%)
Sexual orientation		
Straight or heterosexual	15 (94.7%)	17 (89.5%)
Gay or lesbian	1 (5.3%)	–
Bisexual	–	–
Pansexual	–	–
Asexual	–	–
Queer	–	–
All other sexual orientations	–	–
Prefer to self-describe	–	–
Prefer not to say	3 (15.9%)	2 (10.5%)
English as first language		
Yes	–	9 (47.4%)
No	–	9 (47.4%)
Prefer not to say	–	1 (5.3%)
Last time maternity services were used		
<1 year ago	–	2 (10.5%)
1 year ago	–	2 (10.5%)
2 years ago	–	4 (21.1%)
3 years ago	–	3 (15.8%)
>3 years ago	–	7 (36.8%)
Prefer not to say	–	1 (5.3%)

*Note: Ethnicity and sexual orientation data are reported using the Office for National Statistics census categories.

### Top five themes across all nominal groups

Discussions within each nominal group were indicative of a range of problems associated with maternity care experiences for Black, African, Caribbean and mixed-Black heritage families, such as negative experiences with healthcare professionals, difficulties accessing postnatal support and a lack of adequately culturally adapted information. In total, 89 unique ideas were generated across all nominal groups as solutions, which were coded according to the thematic framework (table 3). The top five co-production themes varied across the nominal groups, although ‘staff knowledge and capabilities’ was common to all. ‘Information and education’ was a shared theme within community and healthcare nominal groups, but notably ranked the highest among the maternity hospital staff, with 60.0% relative importance within that group. ‘Coordination and integration of care’ was important to most professional groups but had a higher relative importance among local authority staff (20.0%), and wider healthcare staff (32.5%), but was not prioritised highly by maternity hospital staff. ‘Advocacy mechanisms’ was considered a key priority within the community nominal groups (15.8% relative importance) but not included in the top five priorities for other groups. The ‘mental health’ theme was ranked fourth in the community nominal groups but was absent across all professional groups (table 5).

**Table 5:** Top five prioritised themes by each nominal group

	Final rank(relative importance %)^*^
Community (public and VCSE sector)	
Staff knowledge and capabilities	29.8
Advocacy mechanisms	15.8
Information and education	13.7
Mental health	13.7
Coordination and integration of care	11.6
Maternity hospital staff	
Information and education	60.0
Staff knowledge and capabilities	11.1
Access to care	10.0
Community partnerships	6.7
Respect for person-centred care	5.6
Wider healthcare staff	
Coordination and integration of care	32.5
Community partnerships	29.2
Staff knowledge and capabilities	15.8
Information and education	10.0
Organisational policies	7.5
Local authority	
Staff knowledge and capabilities	26.7
Addressing ethnicity-based safety risks	21.3
Coordination and integration of care	20.0
Respect for person-centred care	17.3
Organisational policies	5.3

* Please note that the percentages will not sum to 100% as only the top five themes with the highest relative importance are presented.

VCSE, voluntary, community, and social enterprise.

### Alignment of priorities between community members and ICS professionals: qualitative contextualisation

As the ambition of co-producing health services is to align care with patient needs,^18 38^ the following section describes the top five themes ascertained from the community workshops, with similarities and differences between the community and professional nominal groups for these themes, based on framework analysis of workshop meeting notes.

#### Staff knowledge and capabilities

Co-producing effective staff training was important for all nominal groups, with an emphasis on culturally competent care and how staff can better provide inclusive, person-centred care for Black, African, Caribbean and mixed-Black families. Within the community workshops, some attendees expressed the need to engage in ‘code-switching’, in which the style and grammar of language used is adapted based on the conversation’s topic, participants and situation.^39^ This was done to avoid being stereotyped by health professionals, such as assumptions about pain tolerance stemming from ‘strong Black woman’ stereotypes, women perceived to be young mothers; family’s education status or assumptions made about lack of support from the father or partner. In addition, there were concerns that Black, African, Caribbean and mixed-Black families were unknowingly being referred to social services by healthcare professionals at higher rates than other ethnic groups. Effective, in-person training co-produced with lived experience was seen by all workshop attendees as an effective way to address these biases and improve care experiences. Some hospital staff recognised that these biases exist during the discussions, and felt it was important that any mythbusting training used a data-driven approach.

Additionally, members of the public and voluntary sector perceived that poor interpersonal skills of staff contributed to feelings of vulnerability and trauma experienced during care. Unprofessional or condescending language was reportedly used by clinicians, and appropriate explanations or debriefs omitted after traumatic events. It was thus recommended that staff undergo in-person culturally sensitive communication training, including empathetic explanations of medical procedures. All groups acknowledged the system pressures that impact staff behaviours and capabilities, calling for improved staff-to-patient ratios and better staff health and well-being support.

#### Advocacy mechanisms

‘Advocacy mechanisms’ was top five priority only for the community nominal groups. There was a strong focus on developing strategies to support Black, African, Caribbean and mixed-Black heritage families to assert their rights and voice concerns at all maternity care stages. Many individuals felt that they had no voice, even if they knew their rights, as their concerns were often dismissed by healthcare professionals. Proposed solutions included independently created, culturally tailored birthrights online resources and offline education sessions to be promoted by health and local authority public health teams, establishing organisational accountability. Proposed topics for resources included shared decision-making, pain management, bodily autonomy, informed consent and how families can access their medical records. Furthermore, members of the public and VCSE leads also sought greater collaboration with the VCSE sector to generate funding and resources to provide independent community advocates for families when receiving antenatal, intrapartum and postnatal care.

Improving the access, transparency and overall purpose of the complaints procedure within the NHS was important to community members. This was echoed by regional healthcare professionals, who suggested co-developing inclusive and effective mechanisms to hold healthcare organisations to account in reducing ethnic disparities in care and racially discriminatory behaviours.

#### Information and education

Providing information and education for public and service users was highly valued by communities and healthcare staff, and was the most important theme for maternity hospital staff. Community group-proposed solutions included culturally appropriate personalised face-to-face consultations, outside routine antenatal appointments, to guide first-time mothers and their partners through the stages of pregnancy. Community groups wanted VCSE organisations, schools, NHS and local authorities to work together to increase awareness among Black, African, Caribbean and mixed-Black heritage youths on maternity-related issues such as a fertility and family planning, marriage and relationships, parental care and ethnicity-related risk factors (eg, sickle cell haemoglobinopathy).

Increasing consultation duration and restructuring of antenatal care appointments was also suggested by healthcare staff, while acknowledging the system and workforce barriers this may pose. Proposed changes from maternity hospital staff focused mostly on improved information-delivery to families, through regular online question-and-answer forums with health professionals and a review of information presented on hospital website, leaflets and the hospital’s maternity helpline. Relatedly, the availability and quality of translation and interpretation services was raised in all workshops and was prioritised in the community and health professional nominal groups. Improved identification methods for mothers requiring interpretation services were suggested, ensuring interpreter availability at every visit. Discontinuation of funding for qualified bilingual community interpreters trained in maternity care was highlighted, with VCSE attendees emphasising the need to reinstate this funding.

#### Mental health

Solutions to address mental health concerns were raised only within the community nominal groups. Several community members felt they had reduced self-esteem due to trauma experienced or negative interactions with staff during childbirth. To address this, VCSE sector and members of the public wanted integrated, inclusive and personalised mental health support, such as trauma-informed counselling and emotional support, throughout the full maternity pathway. The importance of providing mental health support for husbands and partners was also emphasised, including support for carers around postnatal depression and birth trauma.

#### Coordination and integration of care

Within this theme ‘continuity of care and carer’, having the same midwife at each antenatal care appointment and during childbirth, was a prioritised solution across all nominal groups. However, feasibility constraints were acknowledged given staff shortages and current structure of the healthcare system. To address these constraints, the importance of collaborating with communities and the VCSE sector was highlighted by all attendees as a way of providing gap-filling support to families. Integrated system working was suggested to help service users to navigate referrals between primary, secondary and specialist care (eg, integrating fetal genetic testing with maternity services). Finally, the need to signpost families to holistic, integrated support (eg, housing, economic support, social support networks) was considered vital to addressing the wider determinants of health.

## Discussion

This co-production exercise consulted healthcare professionals, local authorities, the VCSE sector and members of the public from Black, African, Caribbean and mixed-Black heritage backgrounds on how to improve the ethnic inequities in the experience of maternity care within the NWL ICS. By running separate workshops for each group using the NGT method, we elucidated key differences between the priorities of those with lived and community experience, and the belief systems held by health professionals, underscoring the importance of centring lived experience in addressing maternal health ethnic inequalities.

All nominal groups prioritised improving staff knowledge and capabilities in providing culturally sensitive care for Black, African, Caribbean and mixed-Black heritage families. This aligns with national findings in England that negative care experiences for this ethnic group may stem from staff’s poor attitudes, knowledge and racially based assumptions, leading to discrimination and dismissal of their needs.^40 41^ Although all groups prioritised this theme, it held double the relative importance for community and local authority groups compared with health professionals. This may be due to ongoing cultural competency training already undertaken by health professionals, leading to increased awareness of the complexities involved in articulating its mechanisms of change for impact.^42^ However, evidence suggests that initiatives such as cultural competency training do increase patient satisfaction among minoritised ethnic groups, but further studies are required to understand impacts and underlying mechanisms of change,^43^ as well as how organisations can continuously improve culture awareness and skills of their staff.^44^

In terms of diverging priorities between groups, maternity hospital staff prioritised improving the quality of information and education for service users, while community members focused on advocacy mechanisms, addressing organisational cultures and historical mistrust in statutory services. Service users able to self-advocate are more likely to get the care they need, although this varies based on an individual’s confidence, and anticipated repercussions (eg, risks of conflict or mistreatment by the care provider).^45 46^ Accordingly, our findings highlight the need for health and social care professionals to recognise the crucial role of independent community advocates in supporting minoritised ethnic maternity service users.^47^ This may in turn help to promote reporting of formal complaints by Black, African, Caribbean and mixed-Black heritage families, as nationally, 48% of women from this ethnic group do not make a complaint to their NHS provider despite being dissatisfied with care.^41^ Although ensuring the provision of high-quality information and education is important, non-interactive mechanisms such as leaflets or websites may not always ensure that families fully understand and can critically engage with the content. Community advocates can play an important health education role by providing this information in a trusted, culturally appropriate format.

Finally, solutions addressing the mental health of women and their partners were only raised within the community nominal groups. According to the 2024 Mothers and Babies: Reducing Risk through Audits and Confidential Enquiries across the UK (MBRRACE-UK) report, postnatal mental health is remains a key driver of maternal mortality in the UK.^10^ That mental health-related solutions were not discussed in the professional workshops aligns with global evidence on the lack of integration of perinatal mental healthcare in routine health and social care services,^48^ highlighting a potential service improvement opportunity. This is particularly pertinent given that women from Black African groups have significantly lower access to community mental health services than White British women.^9^

### Strengths and limitations

This study’s strength lies in engaging diverse relevant groups across an ICS, including health professionals from primary, secondary, regional and national settings, local authority staff, VCSE sector representatives and local community members from Black, African, Caribbean and mixed-Black heritage backgrounds. Notably, half were previous maternity service users and VCSE leads, who are generally less involved in hospital-led patient feedback events. Community workshops benefited from trauma-informed facilitation by community engagement specialists and lived experience public facilitators, all from Black, African, Caribbean and mixed-Black heritage backgrounds, fostering a safe environment for discussing care experiences, potentially enhancing the quality and depth of the discussion. This inclusive approach aligns with national priorities to enhance the ethnic diversity of voices involved in maternity care and health inequalities service transformation.^18 49^

Use of NGT methodology for structuring multigroup engagement to identify co-production priorities constitutes a further notable study strength.^35^ We present a thematic overview of priorities for each nominal group, enabling a nuanced but holistic understanding of the relative importance of themes for different professional groups, community members and VCSE sector. Moreover, use of a well-established method supports replication by quality improvement practitioners, policymakers and uniquely in this study, communities and the VCSE sector, to ascertain priorities in other regional contexts.

This study focused on the experience of maternity care for Black, African, Caribbean and mixed-Black heritage families within one London locality; therefore, our findings cannot be considered generalisable or transferable to other settings. However, we recommend that the methodology used be replicated by commissioners and quality improvement practitioners in maternity and other specialties as a collaborative approach to addressing ethnic inequities. As all attendees volunteered to attend a workshop, we likely captured the voices of individuals with a particular interest in this topic, including staff that worked in field of health inequities or families that were engaged with health services. Thus, views shared during the workshops may not be representative of all perspectives among health professionals or families of Black, African, Caribbean and mixed-Black heritage backgrounds. In addition, roles and seniority of participating health professionals were not collected due to reduce possibility of re-identification through triangulation using external data sources, and this limits further analysis to understand the types of solutions proposed by profile of the professionals. Furthermore, framework analysis to contextualise ideas generated during the workshops was conducted on notes taken during meetings rather than transcripts. Accordingly, analytical insights are limited to what the notetakers deemed important at the time and may not be exhaustive.

### Future directions

As next steps, we are convening a group of senior managers and directors from the local NHS organisations, local authorities, VCSE and the lived experience facilitators. Attendees will be asked to review the prioritised solutions, discuss resourcing requirements, potential challenges and opportunities to embed proposed solutions within services, with a vote on the overall feasibility and impact of each solution. Through this final exercise, the highly ranked feasible and impactful solutions will be selected for co-production, using an integrated care approach to improve maternity care experiences for Black African, Caribbean and mixed-Black heritage families living within the London locality.

## Conclusion

This collaborative exercise identified co-production priorities using the NGT to address ethnic inequities in maternity care for Black, African, Caribbean and mixed-Black heritage families. Engaging diverse groups using NGT, which included health professionals, local authorities, community members and the VCSE sector, revealed key differences in priorities between professionals and the community, emphasising the need to centre lived experiences in quality improvement. The study underscores the value of an integrated, community-centred approach, promoting collaboration beyond the NHS to reduce maternity care inequities and building community trust in prioritisation exercises by leveraging lived experience expertise.

## Supplementary material

10.1136/bmjqs-2024-017848 online supplemental file 1

10.1136/bmjqs-2024-017848 online supplemental file 2

## Data Availability

All data relevant to the study are included in the article or uploaded as supplementary information.
