# Supplementary material for: Improving the maternity experience for Black, African, Caribbean and mixed-Black families in an integrated care system: a multigroup community and interprofessional co-production prioritisation exercise using nominal group technique
Source: BMJ Qual Saf. 2024 Nov 27;34(5):e017848. doi: 10.1136/bmjqs-2024-017848 (PMC12013574; doi:10.1136/bmjqs-2024-017848)
Supplement: online supplemental file 1 [file bmjqs-34-5-s001.pdf]

Supplementary file 1 - individual workshop ideas and rankings

Supplementary table 1a. *Maternity hospital staff ranked ideas (nominal group 1), N=5*

Ideas generated	Sum of scores (for each idea)	Relative importance (%)*	Ranked priority	Frequency of voting (for each idea)	Ranked priority (via scores & priority)
Improve quality of information provided in 24/7 maternity helpline	20	22.2	1	5	**1**
Availability and quality of translation and interpretation	13	14.4	2	4	**2**
Improve quality of information provided during ANC period	11	12.2	3	3	**3**
Cultural competency & awareness of ethnicity-based risks for staff	10	11.1	4	3	**4**
Earlier uptake of ANC first appointment	9	10.0	5	2	**5**
Regular online Q&As with health providers during ANC period	6				
Service re-design with community groups and service users	5				
Listening and shared decision-making with service users	5				
Improving patient information - website, leaflets, etc.	4				
Involvement of men and partners	3				
Involving volunteer community members to support and advocate for service users	2				
Continuity of care and carer	1				
Collaboration with communities and VCSEs to signpost to services	1				
Increasing length of time and restructuring of ANC appointments	0				
Improve staff knowledge about FGM referral services	0				
Out of hours support when responding to serious incidents	0				

* relative importance (%) = [total score for idea / (number of participants in group x total possible score (i.e. 5+4+3+2+1)]*100

Supplementary table 1b. *Wider NHS staff ranked ideas (nominal group 2), N=8*

Ideas generated	Sum of scores (for each idea)	Relative importance (%)	Ranked priority	Frequency of voting (for each idea)	Ranked priority (via scores & priority)
Continuity of care and carer	19	15.8	1	7	**1**
Staff education - cultural competency & awareness of ethnicity-based risks	19	15.8	1	5	**2**
Collaboration with communities and VCSEs to provide gap-filling support for service users	14	11.7	2	5	**3**
Co-producing services and improvements with service users and communities	12	10.0	3	3	**4**
Availability and quality of translation and interpretation	7	5.8	4	2	**5**
Sustained regular engagement with communities by maternity staff at all levels	6	5.0	5	2	
Focusing maternity care improvements in high deprivation areas	6	5.0	5	2	
Increasing length of time and restructuring of ANC appointments	5				
Working across sectors and strengthening relationships between primary, secondary, and voluntary sector	5				
Smoothen navigation and system for service users across maternity journey	5				
Regional and local organisations creating actions from national recommendations	4				
Continuous ongoing monitoring of patient experience with health inequalities lens	4				
Collaboration with VCSEs to signpost to services	4				
Involving volunteer community members to support and advocate for service users in hospital	4				
Building trust and being honest with communities and service users about what we can/ can't offer	3				
Sharing learning of safety incidents related to Black and other minority ethnic service users	2				
Improve ethnicity data collection in maternity services	1				
Zero tolerance policy for staff treating patients negatively based on race and class	0				
Unconscious bias training for staff	0				
Ethnically diverse workforce including bilingual workers and peer support workers	0				
Address neonatal unit experience for unexpected admissions	0				
Improved use of social media to disseminate information	0				
Increase community networking events (e.g. coffee mornings, baby massage) to understand needs	0				
Develop protocol for people who do not engage in services (e.g. coming only for one scan)	0				
Increase time available for social prescribers to support service users	0				
Co-develop a way to hold the healthcare system to account (not just about fatal or near-misses).	0				
Strengthen role of healthcare organisations as anchor institutions within the community	0				
Improving access to services in ANC period	0				
Better listening to women during labour with ethnicity lens	0				

Supplementary table 1c. *Local authority ranked ideas (nominal group 3), N=5*

Ideas generated	Sum of scores (for each idea)	Relative importance (%)	Ranked priority	Frequency of voting (for each idea)	Ranked priority (via scores & priority)
Cultural competent care training for staff	19	25.3	1	5	**1**
Staff listening to service users and providing patient-centred care	13	17.3	2	4	**2**
Continuity of care and carer	10	13.3	3	3	**3**
Improving clinical safety for mother and baby	9	12.0	4	2	**4**
Organisational prioritisation and embedding of equality, diversity, inclusion values	4	5.3	5	1	**5a**
Make it easier for service users to navigate maternity system	4	5.3	5	1	**5b**
Staff acknowledging vital signs for women as they are	3				
Staff awareness of ethnicity-based risks	3				
Language barriers and translation support	2				
Collaboration with communities and VCSEs to provide additional support for service users	2				
Earlier uptake of ANC first appointment	2				
Improving staff to patient ratio	1				
Understanding risks using certain equipment on specific ethnic groups	1				
Not going to the same communities for co-production	1				
Sustained transformation of services within integrated care system	1				
Improving experience for women who have had IVF abroad	0				

Supplementary table 1d. *Community ranked ideas (nominal group 4), N=10*

Ideas generated	Sum of scores (for each idea)	Relative importance (%)	Ranked priority	Frequency of voting (for each idea)	Ranked priority (via scores & priority)
Face-to-face session talking through stages of pregnancy for your first baby and having resources upfront	31	13.3	1	7	**1**
Supporting mental health of women throughout the maternity journey	19	11.3	2	5	**2**
Creating a birth rights course provided by the NHS for mothers so that they can advocate for themselves.	17	10.0	3	4	**3**
Offer counselling and emotional support during pregnancy	13	9.3	4	5	**4**
Creating paid maternity 'ambassadors' that are trained to provide informal support to women at each maternity stage and support complaints process.	13	8.7	5a	5	**5**
Cultural safety and awareness training for staff (mythbusting)	13	8.7	5b	4	
Resources to train doctors and professionals on communication skills	12				
Having (easier) access to your medical records and knowing how to access this	11				
Offer counselling and emotional support for partners	10				
Supporting staff to better explain with empathy what they are doing medically	9				
Offering holistic support on overall health and not just about my pregnancy or about the baby	7				
Having the same midwife throughout the maternity journey	7				
Postnatal mental health support that is personalised	6				
Training for clinicians and midwives to improve their skills on clinical safety issues	5				
Improving the access and transparency of the complaints procedure (how to complain and how it will be dealt with)	3				
Voluntary sector, schools, NHS working together to provide training for young adults around fertility, marriage, birthing process, parental care, key risk factors (e.g. sickle cell)	2				
Doulas and champions that can support you and advocate for you and your birthing plan	1				
Supporting the health and wellbeing of maternity staff	1				
Training for midwives to spot and support neurodivergent women	0				
Having a mental health team or professional to support both parents	0				
Improving the quality of food offered and making sure food is culturally appropriate	0				
Having a separate role to help women to select their birth partner	0				

Supplementary table 1e. *Community ranked ideas (nominal group 5), N=9*

Ideas generated	Sum of scores (for each idea)	Relative importance (%)	Ranked priority	Frequency of voting (for each idea)	Ranked priority (via scores & priority)
Communication training for staff working with black mothers	21	15.6	1	7	**1**
Clearer communication between staff and service user during medical intervention	16	11.9	2	5	**2**
Co-produced solutions to support black fathers (e.g. tailored antenatal classes, education on emergency procedures, relationships and marriage support, postnatal support)	14	10.4	3	4	**3**
Having the same midwife or team throughout the maternity journey	13	9.6	4	4	**4**
Listening to women and respecting their autonomy	11	8.1	5	3	**5**
Specific training for all staff on how to support mothers that are being perceived as being young	10				
Cultural awareness training for staff	9				
Information about local support networks given during antenatal, especially for new arrivals and translation support	6				
Offer options for infant feeding and supporting breastfeeding or formula feeding	5				
Breastfeeding education in schools and colleges	5				
Using black male NHS staff as role models to support black fathers in a culturally representative way	5				
Fund services to provide interpreters in the NHS that are trained in maternity care	5				
Having independent advocates separate from the NHS to provide support for mums	4				
Using a holistic approach to address women's needs before being discharged	3				
Better ways to identify women with interpretation needs so interpreter is available at each visit	3				
Clearer understanding of where to complain and how the complaint is being dealt with	1				
Staff trained to understand the holistic needs of the mum and what support she needs	1				
