# Supplementary material for: Improving the maternity experience for Black, African, Caribbean and mixed-Black families in an integrated care system: a multigroup community and interprofessional co-production prioritisation exercise using nominal group technique
Source: BMJ Qual Saf. 2024 Nov 27;34(5):e017848. doi: 10.1136/bmjqs-2024-017848 (PMC12013574; doi:10.1136/bmjqs-2024-017848)
Supplement: online supplemental file 2 [file bmjqs-34-5-s002.pdf]

Supplementary file 2 - Ranked themes per nominal group technique workshop

Supplementary table 2a. *Maternity hospital staff ranked themes (nominal group 1)*

Theme	Sum of scores per theme	Relative importance (%)	Ranked priority	Frequency of voting (for each idea)	Ranked priority (via scores & priority)
Information and education	54	60.0	1	6	**1**
Staff interactions	10	11.1	2	2	**2**
Access to care	9	10.0	3	2	**3**
Community partnerships	6	6.7	4	2	**4**
Respect for person-centred care	5	5.6	5	1	**5**
Advocacy	2	2.2		1	
Mental health	0	0.0		0	
Involvement of friends and family	3	3.3		1	
Addressing ethnicity-based safety risks	0	0.0		0	
Organisational policies	0	0.0		0	
Coordination and integration of care	1	1.1		1	

Supplementary table 2b. *Wider NHS staff ranked themes (nominal group 2)*

Theme	Sum of scores per theme	Relative importance (%)	Ranked priority	Frequency of voting (for each idea)	Ranked priority (via scores & priority)
Coordination and integration of care	39	32.5	1	7	1
Community partnerships	35	29.2	2	6	2
Staff interactions	19	15.8	3	3	3
Information and education	12	10.0	4	3	4
Organisational policies	9	7.5	5	4	5
Respect for person-centred care	0	0.0		1	
Advocacy	4	3.3		2	
Mental health	0	0.0		0	
Involvement of friends and family	0	0.0		0	
Access to care	0	0.0		2	
Addressing ethnicity-based safety risks	2	1.7		1	

Supplementary table 2c. *Local authority ranked themes (nominal group 3)*

Theme	Sum of scores (for each idea)	Relative importance (%)	Ranked priority	Frequency of voting (for each idea)	Ranked priority (via scores & priority)
Staff interactions	20	26.7	1	2	**1**
Addressing ethnicity-based safety risks	16	21.3	2	4	**2**
Coordination and integration of care	15	20.0	3	4	**3**
Respect for person-centred care	13	17.3	4	1	**4**
Organisational policies	4	5.3	5	1	**5**
Information and education	2	2.7		1	
Advocacy	0	0.0		0	
Mental health	0	0.0		0	
Involvement of friends and family	0	0.0		0	
Access to care	2	2.7		1	
Community partnerships	3	4.0		2	

Supplementary table 2d. *Community ranked themes (nominal groups 4 and 5)*

Theme	Sum of scores (for each idea)	Relative importance (%)	Ranked priority	Frequency of voting (for each idea)	Ranked priority (via scores & priority)
Staff interactions	85	29.8	1	8	**1**
Advocacy	45	15.8	2	7	**2**
Information and education	40	14.0	3	6	**3**
Mental health	40	14.0	3	5	**4**
Coordination and integration of care	31	10.9	4	6	**5**
Involvement of friends and family	21	7.4	5	3	
Respect for person-centred care	17	6.0		2	
Access to care	0	0.0		0	
Addressing ethnicity-based safety risks	3	1.1		1	
Organisational policies	0	0.0		1	
Community partnerships	0	0.0		0	
